# Quantitative analysis of hydrogen sites and occupancy in deep mantle hydrous wadsleyite using single crystal neutron diffraction

**DOI:** 10.1038/srep34988

**Published:** 2016-10-11

**Authors:** Narangoo Purevjav, Takuo Okuchi, Naotaka Tomioka, Xiaoping Wang, Christina Hoffmann

**Affiliations:** 1 Institute for Planetary Materials, Okayama University, Misasa, Tottori 682-0193, Japan; 2 Kochi Institute for Core Sample Research, Japan Agency for Marine-Earth Science and Technology, Nankoku, Kochi 783-8502, Japan; 3 Chemical and Engineering Materials Division, Neutron Sciences Directorate, Oak Ridge National Laboratory, Oak Ridge, TN 37831, USA

## Abstract

Evidence from seismological and mineralogical studies increasingly indicates that water from the oceans has been transported to the deep earth to form water-bearing dense mantle minerals. Wadsleyite [(Mg, Fe^2+^)_2_SiO_4_] has been identified as one of the most important host minerals incorporating this type of water, which is capable of storing the entire mass of the oceans as a hidden reservoir. To understand the effects of such water on the physical properties and chemical evolution of Earth’s interior, it is essential to determine where in the crystal structure the hydration occurs and which chemical bonds are altered and weakened after hydration. Here, we conduct a neutron time-of-flight single-crystal Laue diffraction study on hydrous wadsleyite. Single crystals were grown under pressure to a size suitable for the experiment and with physical qualities representative of wet, deep mantle conditions. The results of this neutron single crystal diffraction study unambiguously demonstrate the method of hydrogen incorporation into the wadsleyite, which is qualitatively different from that of its denser polymorph, ringwoodite, in the wet mantle. The difference is a vital clue towards understanding why these dense mantle minerals show distinctly different softening behaviours after hydration.

A portion of water in Earth’s oceans is continuously transported into the deep mantle as hydroxyl groups within dense hydrous minerals^1^,^2^,^3^. These minerals are carried by the downward motion of subducting oceanic plates, which are driven by plate tectonics. The mantle transition zone (MTZ) is between 410 km and 660 km depth and has been considered the primary destination for water transported from the oceans^4^,^5^,^6^,^7^. The MTZ consists mainly of wadsleyite and ringwoodite mineral phases, which are the two denser high-pressure polymorphs of olivine^8^. These three phases of (Mg, Fe^2+^)_2_SiO_4_ in the mantle are all nominally anhydrous; however wadsleyite and ringwoodite are capable of incorporating up to approximately 3 wt.% of H_2_O within their crystal structures^3^,^7^,^9^,^10^,^11^,^12^,^13^. To accept such an amount of water while maintaining dense crystal structures, hydrogen cations (H^+^) have been shown to substitute magnesium (Mg^2+^) in both wadsleyite and ringwoodite, and also silicon (Si^4+^) in the case of ringwoodite^11^,^14^,^15^,^16^,^17^.

Previous seismological studies proposed that some regions of the uppermost MTZ, at approximately 410 km depth, had anomalously lower velocities, which were interpreted as a result of the hydration reaction in a wet mantle environment where water transported from the surface became concentrated^5^,^18^. At this depth, olivine transforms into wadsleyite, and is then able to be hydrated. A more recent seismological study proposed that a large region of the lowermost MTZ, around 660 km depth, is so extensively hydrated that it is capable of inducing dehydration melting^19^.

On the other hand, it has commonly been reported by laboratory experiments and first principle calculations that the elastic constants of wadsleyite and ringwoodite monotonically decrease with increasing water concentration^15^,^20^,^21^,^22^. This means that the crystals of wadsleyite and ringwoodite become more compressible after hydration, while their modified spinel and cubic spinel crystal structures, respectively, are preserved^11^,^14^. The structures of both minerals are very similar because they share the cubic closest packing arrangement of oxygen atoms. As mentioned above, the regions comprised of hydrated wadsleyite or ringwoodite crystals with higher compressibility can be seismologically discriminated by their lower velocities, at least in the uppermost or lowermost MTZ, whereas water concentrations in these regions remain very difficult to estimate. This difficulty is partly because the degree of hydration-induced elastic constant reduction is strongly dependent on whether Mg^2+^, Fe^2+^ or Si^4+^ cations exchange with hydrogen during the process^15^,^22^. It has not yet been determined what fraction of these cations can be exchanged for hydrogen, especially in the case of hydrous wadsleyite. Additionally, the elastic constants of wadsleyite and ringwoodite have different sensitivities to the unit concentration of water, even though the two phases have very similar structures in dry conditions^23^. This difference implies that the two mineral phases may have distinctly different mechanisms for exchange between hydrogen and the other cations. In other words, the chemical bonding in hydrous wadsleyite and ringwoodite may be weakened at different positions in their crystal structures. To observe this difference, the hydrogen site and occupancy of wadsleyite was analysed in this study by neutron time-of-flight (TOF) single-crystal Laue diffraction^24^. As demonstrated in this study, this is by far the most powerful structural probe for detecting hydrogen in crystals. It has been successfully applied here for the first time to reveal the nature of chemically-bonded hydrogen in deep Earth minerals.

## Results and Discussion

### Hydrogen Site and Occupancy

**Figure 1:**
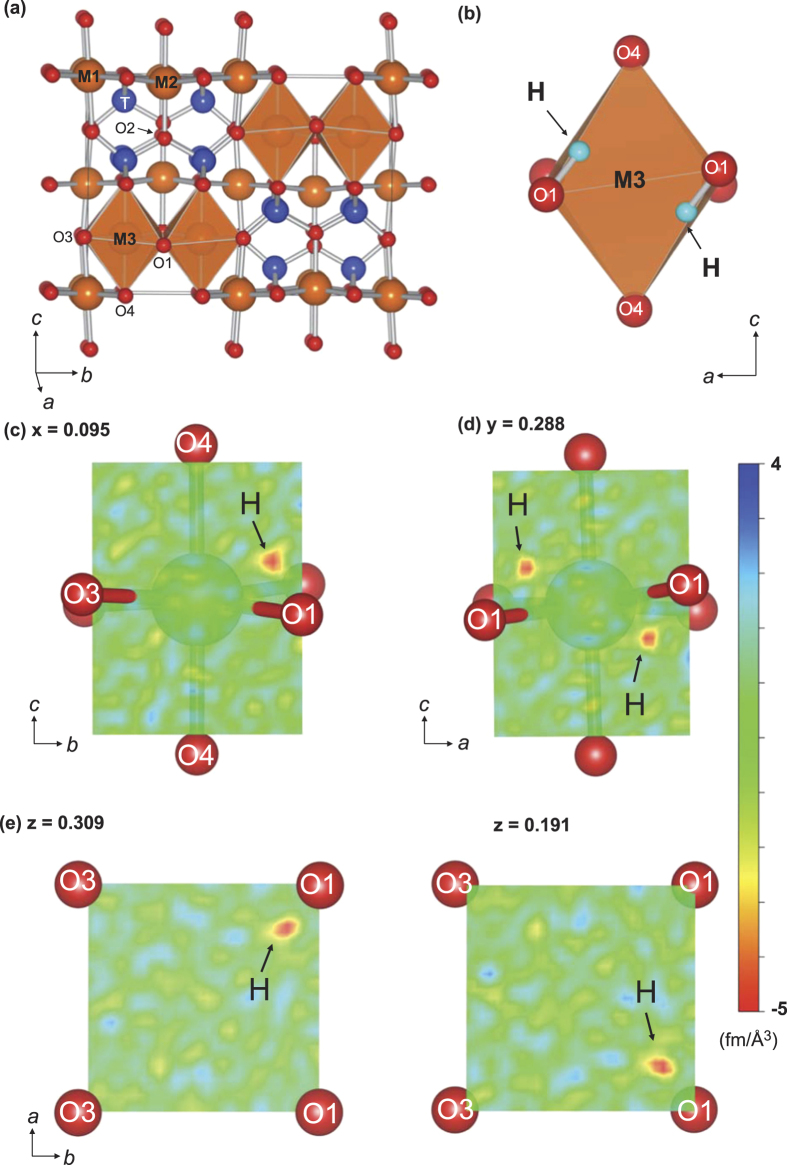
Framework of wadsleyite crystal structure and observed difference Fourier maps by neutron diffraction. These crystallographic illustrations were created using the software VESTA3^51^. (**a**) Dry wadsleyite crystal structure with orthorhombic symmetry (*Imma*). (**b**) Position of H^+^ in the local structure around the M3 octahedral site. The Fourier maps were generated from the difference of scattering length densities between those calculated from the dataset at *d*_*min*_ = 0.30 Å and those simulated for the dry wadsleyite structure. These maps show the slices at (**c**) (100) section at *x* = 0.095, (**d**) (010) section at *y* = 0.288 and (**e**) (001) section at *z* = 0.309 and 0.191, respectively.

Figure 1a shows the crystal structure of anhydrous wadsleyite in the orthorhombic space group *Imma*, which is briefly described here before discussing the hydrogen cations in this framework. Each Mg^2+^ is at one of three specific octahedral sites (M1 to M3), which is surrounded by six O^2−^. Each Si^4+^ is at one specific tetrahedral site (T), which is surrounded by four O^2−^. Additionally, each O^2−^ is at one of four specific sites (O1 to O4), each of which possesses a different geometry of surrounding cations. Among them, O1 is particularly unique because it is not connected to Si^4+^, while all of the others (O2, O3 and O4) have one or two neighbouring Si^4+^.

The structure parameters of wadsleyite obtained from single crystal neutron diffraction are shown in Tables 1 and 2. All atomic positions, including hydrogen, were refined using anisotropic Debye-Waller factors for our experimental hydrous wadsleyite using a high resolution dataset (*hkl*) up to a minimum *d*-spacing (*d*_min_) of 0.30 Å. This resolution was significantly better than that of the previously obtained neutron powder diffraction pattern for a deuterated wadsleyite sample (*d*_min_ = 0.96 Å)^16^. Thus, using our single-crystal dataset, we provided a much more reliable site position and occupancy of hydrogen, as schematically illustrated in Fig. 1b. High-contrast and high-spatial resolution difference Fourier maps were also obtained to help visualise the position of hydrogen in the space-averaged structure (Fig. 1c–e).

The refined result of our dataset showed that there were four equivalent H^+^ positions surrounding each of four equivalent O1 positions in the unit cell. While there was a total of 16 equivalent H^+^ sites, it was found that these sites could not be simultaneously occupied. It was determined that to maintain local charge balance, the two H^+^ sites belonging to one empty M3 octahedron must be simultaneously occupied, where the octahedron consists of six oxygen atoms (two O1, two O3 and two O4). Each of these two H^+^ was located along one of the two edges between the two O1-O4 pairs in that octahedron (Fig. 1b). The refined bond length of O1–H was 0.999(5) Å at 100 K and 0.987(6) Å at 295 K. The difference between these O–H distances is well below the 3-sigma range and they are therefore nominally the same within their standard deviations. Compared with the deuterated wadsleyite, the positions of O1 and H are shifted slightly by 0.001 Å and 0.007 Å, respectively, in the same direction along the *c*-axis^16^. Our observed O–H bond length was 0.05 Å shorter than that of the O–D reported in the deuterated wadsleyite. The hydrogen bond lengths (O4…H) were 2.089(5) Å and 2.105(6) Å at 100 K and 295 K, respectively, where the latter is slightly longer than the former. The hydrogen bonding was almost linear, with an O–H…O angle of 171° at both temperatures. The O4…H hydrogen bond was 0.06 Å longer than that of O4…D in the deuterated wadsleyite, implying that the hydrogenated M3 octahedron had a slightly larger volume than that of the deuterated M3 octahedron.

**Figure 2:**
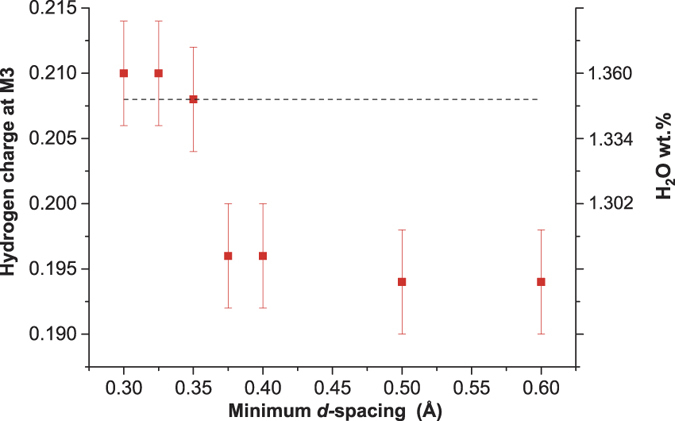
Observed total charge of hydrogen at the M3 octahedral site as a function of *d*_*min*_. The dashed line is the charge of H^+^ independently estimated from the lattice constant ratio *b*/*a*^25^.

The difference Fourier maps show the position of the largest negative scattering length density. The density was −5.3 fm/Å^3^, which was far greater than the other negative residuals observed throughout all the datasets from *d*_min_ = 0.30 Å to *d*_min_ = 0.60 Å (see Fig. S1 for more detail on these residuals). Other than this H site, no other excess scattering length density distribution was observed. Therefore, this H site should be the only one possible place that incorporates protons in the hydrous wadsleyite crystal. To thoroughly establish this fact from a different viewpoint, we made additional efforts to refine the hydrogen occupancy. We compared the occupancy of the H^+^ site refined for several different datasets with the variable *d*_*min*_. Figure 2 shows the charge of H^+^ at the M3 site as a function of *d*_*min*_, which was calculated from the refined occupancies deduced from the datasets of variable *d*_*min*_. Where the *d*_*min*_ was smaller than 0.325 Å, the observed charge of hydrogen became constant, whereas where the *d*_*min*_ was larger than 0.35 Å, the charge depended on *d*_*min*_. The amount of H^+^ on the M3 site was converged to a partial occupancy of 0.105(2). The sum of the charge of Mg^2+^ and H^+^ at M3 at *d*_*min*_ ≤ 0.325 Å was 2.000(4), which is equal with its stoichiometric value. Thus, the constant charge of H^+^ with *d*_*min*_ at 0.30 Å to 0.325 Å at 100 K was proven to be reliable.

The total charge of H^+^ in the bulk wadsleyite crystal can be estimated using the pre-established relationship between the water concentration (determined by infrared spectroscopy and secondary ion mass-spectroscopy), and the lattice constant ratio *b*/*a* (determined by X-ray diffraction)^25^. The total H^+^ charge estimated in this manner from the *b/a* of our wadsleyite sample matched exactly the value deduced from the refined hydrogen occupancies found via neutron diffraction at 100 K. Therefore, all hydrogen cations in the crystal (which collectively affect *b*/*a*) should have been fully concentrated into the M3 cavity. In other words, we confirmed that there are no other hydrogen sites. The maximum allowable concentration of H^+^ in wadsleyite is thus constrained by its full capacity into M3, which corresponds to 3.33 wt.% of H_2_O, as proposed by previous crystal chemical and secondary-ion mass spectroscopy analyses^10^,^26^.

A recent study evaluating the chemical environment of H^+^ in wadsleyite by nuclear magnetic resonance spectroscopy reported that some minor H^+^ was bonded with oxygen O3 that belonged to Si^4+^ 27. This finding is different from our results and may have resulted from different synthesis conditions. While their wadsleyite samples were synthesised in a solid state condition at relatively low temperatures from 1100 °C to 1200 °C, our crystals were grown in a partially-molten silicate melt at 1400 °C (see Methods for details). The strain environment may have also been different, as our single crystal was grown under a hydrostatic condition in the melt, while the growth conditions of their powder samples are unknown. Accordingly, it is necessary to consider the effect of non-hydrostatic growth environments on the oxygen species, which were found to be bonding with a minor fraction of the hydrogen. Additionally, hydrogen may also couple with Fe^3+^ to exchange for Si^4+^ at T sites^28^,^29^,^30^. Further neutron diffraction study is required to observe this cation-coupled substitution mechanism in iron-bearing hydrous wadsleyite crystals at elevated oxygen-fugacity conditions.

**Figure 3:**
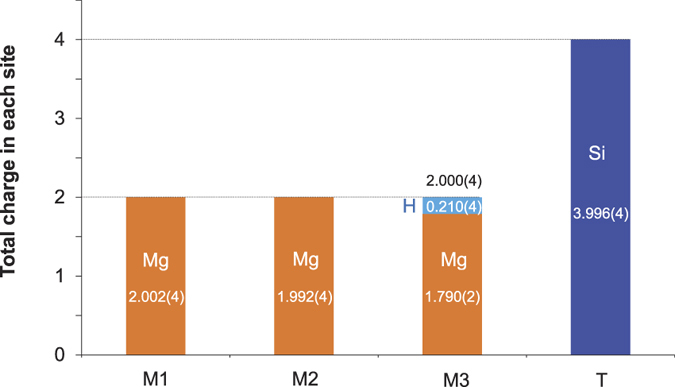
Observed charge number of all cations at each site of the hydrous wadsleyite, calculated from the refined occupancies. These occupancies were not constrained during the structure refinement. The dashed line shows the stoichiometric charge values of each site.

The total charge balances including all cations of the structure are summarized in Fig. 3, which shows all cation species refined at all sites for the dataset at *d*_*min*_ = 0.30 Å obtained at 100 K. The charges of Mg^2+^ and Si^4+^ at M1, M2 and T sites were all equal with their stoichiometric values within the standard deviation of the refinement. The Mg^2+^ charge at M3 was significantly smaller than the stoichiometric value, whereas the sum of Mg^2+^ and H^+^ charges at M3 was equal to the stoichiometric value. The calculated H_2_O concentration from the H^+^ charge at M3 was 1.36 wt.%. Recently, we showed that a powder neutron diffraction study is useful to quantitatively constrain D_2_O concentration in a dense hydrous magnesium silicate^31^. The present study further expanded the unique potential of the neutron diffraction method for a quantitative analysis of H_2_O concentrations of minerals, even at such low concentrations as those found in nominally anhydrous high-pressure minerals.

### Hydrous Wadsleyite and Hydrous Ringwoodite

Seismic velocity in the deep mantle shows major discontinuities at 410, 520 and 660 km, which are ascribed to the occurrence of three phase transformations, including those from olivine to wadsleyite, from wadsleyite to ringwoodite and from ringwoodite to bridgmanite [(Mg, Fe^2+^)SiO_3_] and magnesiowüstite [(Mg, Fe^2+^)O], respectively^8^,^32^. Wadsleyite and ringwoodite in the MTZ are unique with respect to their larger water capacity compared to other nominally anhydrous minerals outside of the MTZ. The lower MTZ is considered to be extensively hydrated, at least in certain regions, as demonstrated by a recent discovery of natural hydrous ringwoodite^7^. Assuming that this water has been transported from the oceans, it would have passed through the upper MTZ and hydrated any wadsleyite in its path.

**Figure 4:**
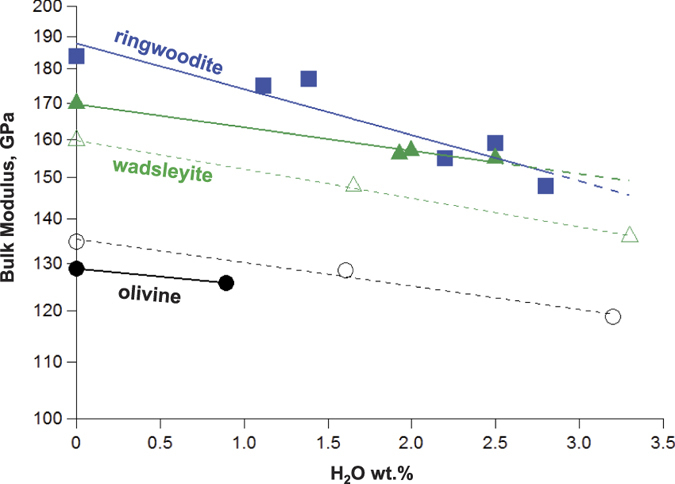
Experimental and theoretical results showing decreasing bulk moduli as a function of increasing water concentration. Triangles, squares and circles represent wadsleyite, ringwoodite and olivine results, respectively. All filled symbols represent experimental results^20^,^21^,^23^,^33^,^34^,^35^,^36^,^37^,^38^,^39^,^40^,^41^. The open triangles and open circles represent density functional theory results for olivine and wadsleyite, respectively, where all hydrogen cations were positioned at M sites by exchanging for Mg^2+^ 15,42. The vertical axis is shown in the log scale. The slopes of decreasing bulk moduli of wadsleyite and olivine are almost comparable, while that of ringwoodite is steeper than these two. See discussion in the text.

As an example of the systematic differences in the properties of wadsleyite and ringwoodite induced after hydration, we summarised the decreasing trends of their bulk moduli as a function of water concentration (Fig. 4). All of these data points were taken from previous experimental and theoretical studies on Fe-free and Fe-bearing hydrous wadsleyite and ringwoodite^15^,^20^,^21^,^23^,^33^,^34^,^35^,^36^,^37^,^38^,^39^,^40^. For reference, we also included experimental and theoretical results for hydrous olivine, which is expected to have a similar hydration mechanism with that of wadsleyite^41^,^42^. It is clear that the slope of decreasing bulk moduli exhibited by ringwoodite is relatively steeper than those of wadsleyite and olivine. Therefore, despite wadsleyite and ringwoodite having very similar crystal structures in their dry forms, they are expected to have significantly different hydration mechanisms. In this study, we have clarified that H^+^ in wadsleyite is exclusively present at one of its octahedral sites (M3), while we previously demonstrated that H^+^ in ringwoodite should be present at both octahedral and tetrahedral sites^17^. This contrast in hydration mechanisms may be the primary reason that a larger reduction of bulk modulus is seen for ringwoodite than for wadsleyite (the two bulk moduli have a 15 GPa difference at zero H_2_O concentration but become indistinguishable at greater than 2.0 wt.% H_2_O). The larger effect of hydration on ringwoodite than on wadsleyite was supported by a previous first principle study, where it was predicted that hydration of the tetrahedral sites is much more effective at reducing elastic properties than hydration of the octahedral sites^22^. Future detailed crystal structure analyses of hydrated mantle minerals would enable more precise predictions of their physical properties, which would lead to a more complete interpretation of the seismic structure of the deep Earth interior.

## Methods

### Sample Preparation

Iron-free single-crystals of hydrous wadsleyite were synthesised by the slow-cooling method using a scaled-up Kawai-type cell^43^. We prepared a mixture of MgO, Mg(OH)_2_ and SiO_2_ powders involving 15 wt.% of H_2_O. About 45 mg of the mixture were sealed into an Au capsule with a 4 mm outer diameter, compressed to 17 GPa pressure and heated to 1380 ± 20 °C for 6 hours, where crystals were grown and homogenised in a silicate partial melt. Over 20 product crystals, all of which had identical chemical compositions^43^, were simultaneously quenched and recovered to ambient conditions. Some of the largest crystals were analysed using microfocus X-ray diffraction (Rigaku RINT RAPID II) for phase identification and several crystals were further analysed by X-ray precession photography for evaluation of crystal quality. A portion of the other crystals were polished and measured by a scanning electron microscope (JEOL JSM-7001F) to confirm their chemical homogeneity. A single crystal was analysed by Fourier-transform infrared (FTIR) spectroscopy (Jasco FTIR-6200) to confirm its homogeneous hydrogen distribution (Fig. S2). Other crystals were ground into fine powder and measured by powder X-ray diffraction (Rigaku SmartLab) at 295 K to precisely refine the lattice constants and the unit cell volume, which were *a *= 5.6865(4) Å, *b *= 11.515(2) Å, *c *= 8.2545(7) Å, *V *= 540.52(5) Å^3^ and *β* = 89.985(13)°. Because the structure of hydrous wadsleyite may show either monoclinic or orthorhombic symmetry^44^,^45^, the *β* angle was determined to define the symmetry of the atomic positions. The *β* angle was almost within one sigma range of 90°, indicating orthorhombic symmetry. We verified that forbidden reflections for the orthorhombic space group *Imma* were not observed in the powder X-ray pattern, providing further support for the orthorhombic symmetry. The refinement of a well-calibrated powder X-ray pattern is the most accurate method for obtaining these constants, even if high-quality neutron diffraction data are available^17^,^31^. Using the results of precession photography, we selected the crystal with the highest crystallinity for neutron diffraction, measuring 800 μm in its longest dimension and completely free from twinning, inclusion and diffuse scattering (Fig. S3).

### Single-Crystal Neutron Diffraction

The diffraction experiment was conducted on the neutron TOF single-crystal Laue diffractometer TOPAZ installed at Spallation Neutron Source (SNS), Oak Ridge National Laboratory (ORNL), USA. This state-of-the-art diffractometer was used successfully to obtain a dataset with a superior spatial resolution, even for the tiny crystal sample volume of this study, which was one order of magnitude smaller than that of crystals used previously for single-crystal neutron diffraction^46^,^47^. The crystal was mounted on a 500 μm-diameter pin-base polyimide tube using a small amount of epoxy resin under a microscope setup. Each dataset (at 100 K and 295 K temperatures) was obtained over a duration of two or three days.

We again verified that neutron intensities of the forbidden reflections for the space group *Imma* were all within the background level. The neutron datasets were then analysed using the GSAS program^48^. The initial non-hydrogen atomic coordinates were taken from a previous powder neutron diffraction study^16^. The initial hydrogen positions were taken from the results of difference Fourier synthesis. The structure refinements were carried out with a secondary type I Lorentzian spread extinction model^49^,^50^, where the *Rw*(F) for the 100 K dataset was 5.1% before and 4.4% after positioning of hydrogen atoms. Thus the full refinement was successfully converged at *Rw*(F) = 4.4% and *R*(F) = 5.8% for the 100 K dataset and at *Rw*(F) = 4.4% and *R*(F) = 6.2% for the 295 K dataset. The Debye-Waller factors of atoms at 100 K were substantially smaller than those at 295 K. This is because thermal vibration of these atoms decreased with decreasing temperature, resulting in a more concentrated scattering length density distribution in space. In the case of hydrogen atoms, such concentration proved to be significant enough to improve the accuracy of the observed scattering length density and site occupancy of hydrogen.

## Additional Information

**How to cite this article**: Purevjav, N. *et al*. Quantitative analysis of hydrogen sites and occupancy in deep mantle hydrous wadsleyite using single crystal neutron diffraction. *Sci. Rep.* **6**, 34988; doi: 10.1038/srep34988 (2016).

## Supplementary Material

Supplementary Information

## Figures and Tables

**Table 1:** Structure parameters at 100 K.

Atom	*x/a*	*y/b*	*z/c*	Occupancy	*U*_11_ × 10^2^*	*U*_22_ × 10^2^*	*U*_33_ × 10^2^*	*U*_12_ × 10^2^*	*U*_13_ × 10^2^*	*U*_23_ × 10^2^*
Mg1	0	0	0	1.001(2)	0.764(12)	0.451(8)	0.942(10)	0	0	0.146(7)
Mg2	0	1/4	0.97043(4)	0.996(2)	0.581(1)	0.393(7)	0.408(7)	0	0	0
Mg3	1/4	0.12444(2)	1/4	0.895(1)	0.451(7)	0.831(7)	0.492(6)	0	−0.018(6)	0
Si	0	0.12039(2)	0.61624(3)	0.999(1)	0.375(7)	0.354(6)	0.368(6)	0	0	−0.011(5)
O1	0	1/4	0.22166(4)	1	0.416(9)	0.641(8)	0.708(8)	0	0	0
O2	0	1/4	0.71653(3)	1	0.591(9)	0.444(7)	0.404(7)	0	0	0
O3	0	0.98829(2)	0.25585(3)	1	0.591(7)	0.547(5)	0.463(5)	0	0	0.049(4)
O4	0.26055(3)	0.12332(1)	0.99392(2)	1	0.452(4)	0.495(3)	0.509(4)	−0.012(4)	0.031(3)	0.001(3)
H	0.0949(1)	0.2882(4)	0.3086(6)	0.105(2)	4.00(26)	3.42(20)	2.87(19)	−0.92(17)	−0.42(20)	−0.79(15)

^*^U_*ij*_ is anisotropic Debye-Waller factors.

**Table 2:** Structure parameters at 295 K.

Atom	*x/a*	*y/b*	*z/c*	Occupancy	*U*_11_ × 10^2^*	*U*_22_ × 10^2^*	*U*_33_ × 10^2^*	*U*_12_ × 10^2^*	*U*_13_ × 10^2^*	*U*_23_ × 10^2^*
Mg1	0	0	0	1.000(2)	0.983(15)	0.598(10)	1.186(13)	0	0	0.111(8)
Mg2	0	1/4	0.97011(5)	0.997(2)	0.802(13)	0.540(9)	0.612(10)	0	0	0
Mg3	1/4	0.12437(3)	1/4	0.898(1)	0.598(9)	1.030(8)	0.724(8)	0	−0.058(7)	0
Si1	0	0.12042(3)	0.61613(4)	1.001(2)	0.503(8)	0.478(7)	0.494(8)	0	0	−0.015(6)
O1	0	1/4	0.22140(5)	1	0.538(11)	0.807(9)	0.900(11)	0	0	0
O2	0	1/4	0.71627(4)	1	0.824(11)	0.550(8)	0.548(9)	0	0	0
O3	0	0.98818(2)	0.25595(3)	1	0.742(8)	0.712(6)	0.627(7)	0	0	0.093(5)
O4	0.26076(3)	0.12330(2)	0.99382(2)	1	0.583(5)	0.653(4)	0.699(5)	−0.014(5)	0.067(4)	−0.003(3)
H	0.0933(2)	0.2873(5)	0.3076(8)	0.081(2)	3.42(32)	2.56(20)	2.04(23)	−0.73(19)	−0.13(22)	−0.58(18)

^*^U_ij_ is anisotropic Debye-Waller factors.
