# Supplementary material for: Quantitative analysis of hydrogen sites and occupancy in deep mantle hydrous wadsleyite using single crystal neutron diffraction
Source: Sci Rep. 2016 Oct 11;6:34988. doi: 10.1038/srep34988 (PMC5057097; doi:10.1038/srep34988)
Supplement: Supplementary Information [file srep34988-s1.pdf]

Supplementary information

Quantitative analysis of hydrogen sites and occupancy in deep mantle hydrous wadsleyite using single crystal neutron diffraction

Narangoo Purevjav[1]*, Takuo Okuchi[1], Naotaka Tomioka[1,2]

Xiaoping Wang[3], and Christina Hoffmann[3]

[1]Institute for Planetary Materials, Okayama University, Misasa, Tottori 682-0193, Japan.

[2]Kochi Institute for Core Sample Research, Japan Agency for Marine-Earth Science and Technology, Nankoku, Kochi 783-8502, Japan.

[3]Chemical and Engineering Materials Division, Neutron Sciences Directorate, Oak Ridge National Laboratory, Oak Ridge, TN 37831, USA.

*Corresponding author: pxec5a1c@s.okayama-u.ac.jp

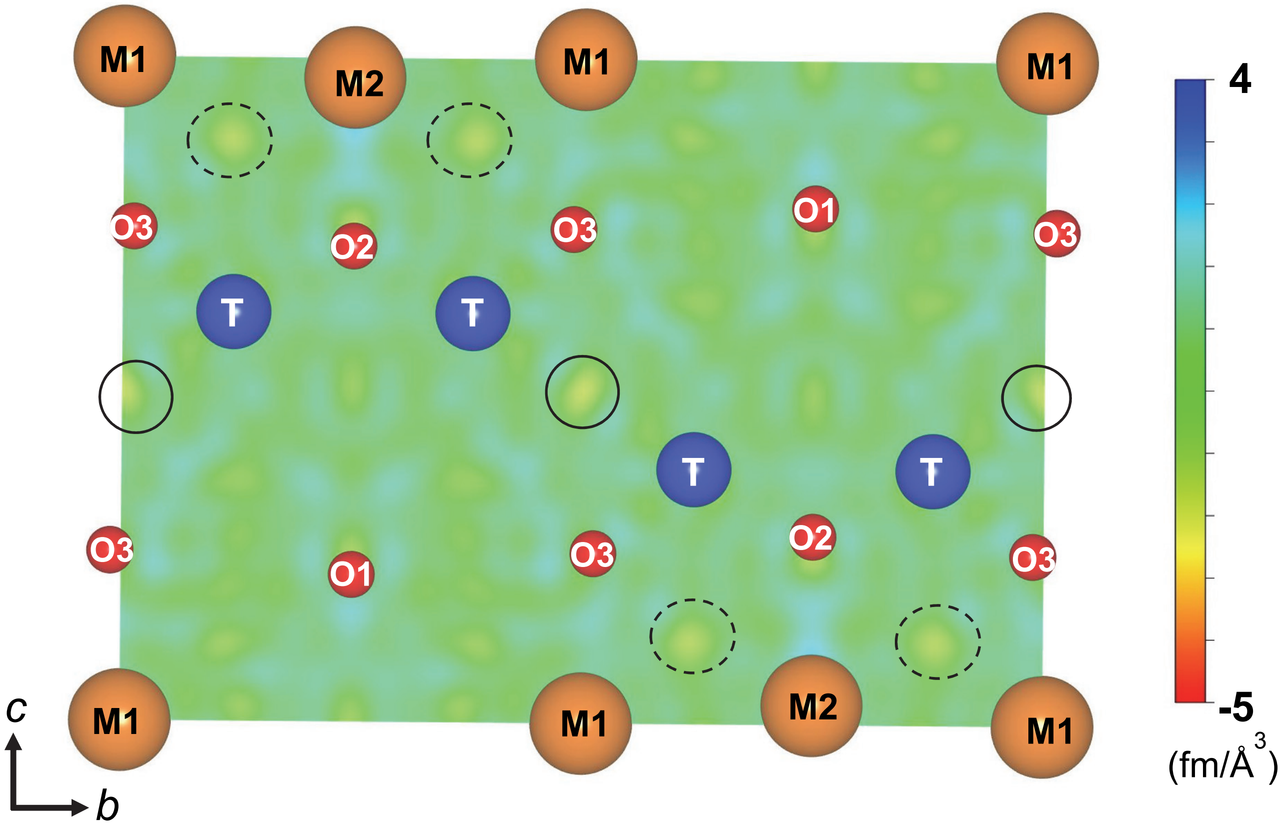

Fig. S1. The difference Fourier map showing the positions of the other negative residuals. The map was generated from the difference of scattering length densities between the dataset with d_{min} = 0.6 Å and the refined structure model including the hydrogen. The solid and dashed circles are the positions of the residuals, which remained throughout all the difference Fourier maps constructed from several datasets with variable d_{min} from 0.30 Å to 0.60 Å. The other residuals are highly fluctuating as the d_{min} of the dataset was changing. The dashed circle at -1.7 fm/Å^3 is positioned at (x = 0; y = 0,6161; z = 0,1199). The solid circle at -2.0 fm/Å^3 is positioned at (x = 0,5; y = 0.0120; z = 0.0162). We consider that these residuals cannot be due to hydrogen, because the distances from these to their closest oxygen anions are 1.64 Å and 1.96 Å respectively, which are too long distances to form covalent chemical bonding.

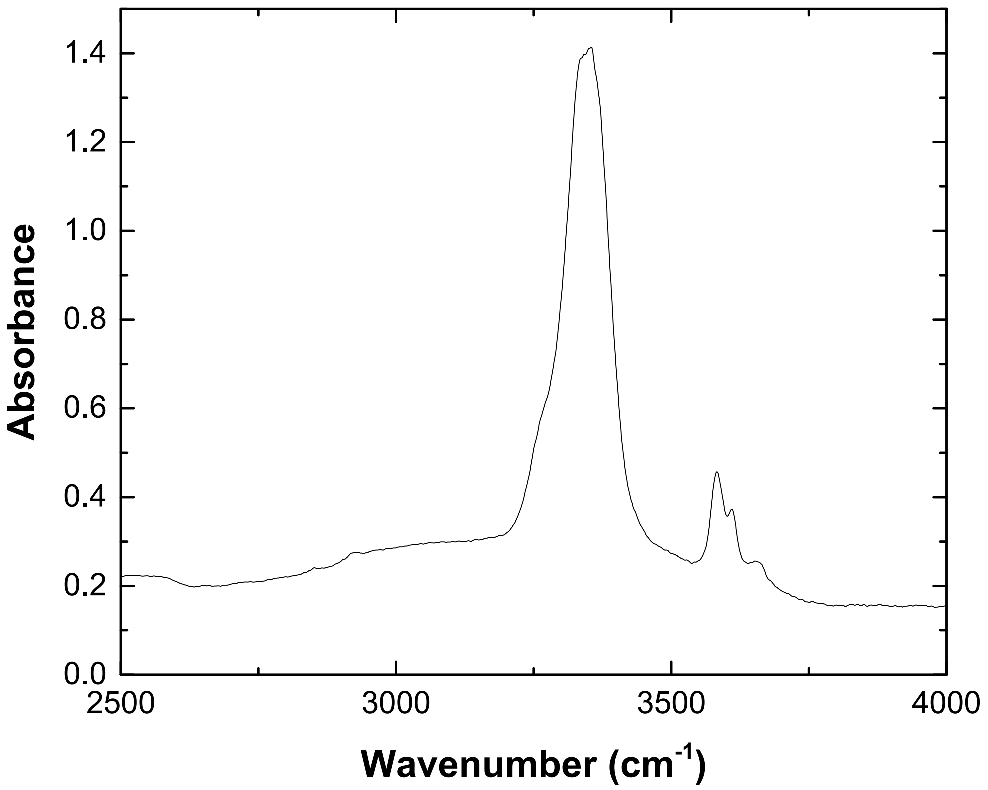

Fig. S2. A representative FTIR spectrum of the hydrous wadsleyite crystal. A few other spectra were also measured to confirm homogeneous distribution of hydrogen.

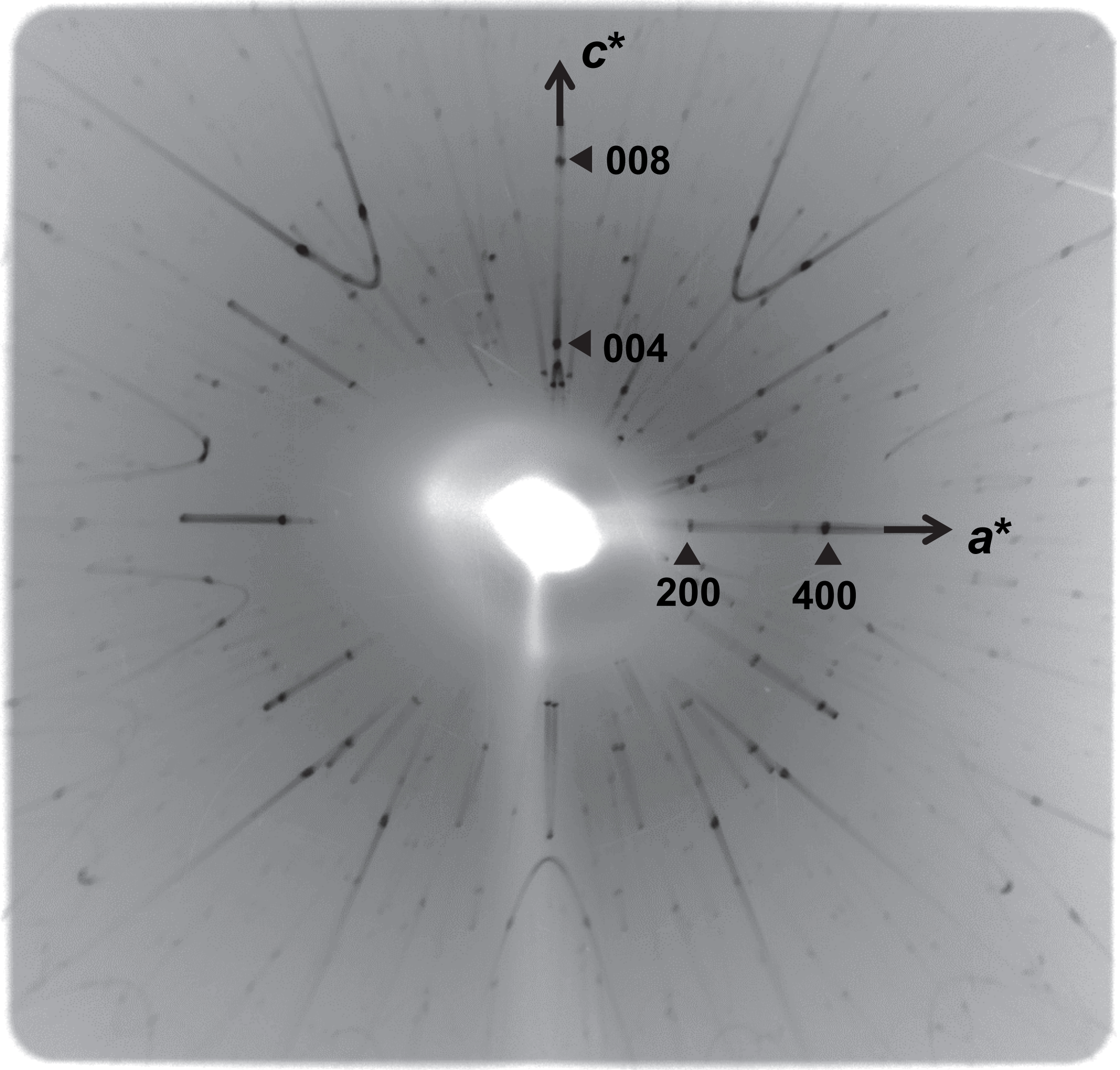

Fig. S3. The X-ray precession photograph of the hydrous wadsleyite crystal subjected for single-crystal neutron diffraction. The numbers denote Miller indices of diffraction spots indicated by the triangles.
